# Risk factors for complications after infantile enterostomy: development of a clinical prediction model

**DOI:** 10.3389/fpubh.2025.1566789

**Published:** 2025-07-03

**Authors:** Zhaolan Zeng, Yanling Hu, Shulin Hou, Ru Yang, Zeyao Shi, Xiaowen Li

**Affiliations:** ^1^Department of Neonatology Nursing, West China Second University Hospital, Sichuan University, Chengdu, China; ^2^Key Laboratory of Birth Defects and Related Diseases of Women and Children (Sichuan University), Ministry of Education, Chengdu, China

**Keywords:** infant, enterostomy, complication, risk factor, nomogram prediction model

## Abstract

**Background:**

Enterostomy is a common surgical procedure for treating acute abdomen in infants. However, the associated complication incidence is high, which significantly impacts infants’ recovery. This study aimed to identify risk factors of short-term complications and develop a prediction model in infants with enterostomy.

**Methods:**

We retrospectively analyzed the clinical data of infants who underwent enterostomy at Sichuan University West China Second Hospital from November 2021 to June 2024. Multifactorial logistic regression analysis was used to screen the risk factors for postoperative complications related to enterostomy in infants, and R software was applied to develop a nomogram prediction model. The accuracy and clinical utility of the prediction model were verified by the receiver operating characteristic (ROC) curve, calibration curve, and decision curve analysis (DCA).

**Results:**

A total of 155 infants were included in this study, with 61 cases (39.35%) in the complication group and 94 cases (60.65%) in the non-complication group. Multifactorial logistic regression analysis showed that smaller weight at surgery (OR = 0.999, 95% CI: 0.999 ~ 1.000, *p* = 0.044), small intestine stoma (OR = 6.405, 95% CI: 1.647 ~ 24.916, *p* = 0.007), and prolonged duration of postoperative high-level C-reactive protein (CRP) (OR = 1.081, 95% CI: 1.001 ~ 1.167, *p* = 0.048) were independent risk factors for complications related to enterostomy in infants. The area under the curve (AUC) of the risk prediction model was 0.784 (95% CI: 0.712 ~ 0.857), and the goodness-of-fit test value of the Hosmer-Lemeshow was 0.604, higher than 0.05, indicating that the regression model had a significant fitting effect. The calibration curves and DCA demonstrated high predictive value and clinical efficiency.

**Conclusion:**

The smaller weight at surgery, small intestine stoma, and longer duration of postoperative high-level CRP may be used to identify the risk of short-term complications after enterostomy. This prediction model is provided for medical staff to evaluate complication-associated risk and take measures for those infants at risk.

## 1 Introduction

Temporary enterostomy serves as a crucial surgical procedure for treating neonatal surgical diseases such as necrotizing enterocolitis (NEC) (1), Hirschsprung disease (2), congenital anal atresia (3), and intestinal obstruction (4). Unlike permanent stomas in adults, infant enterostomies are primarily temporary therapeutic measures, with stoma reversal generally performed 5–22 weeks after enterostomy formation (5, 6). Although enterostomy can significantly reduce perioperative mortality, its high complication rate (17–68%) remains a severe challenge (3, 7, 8). Owing to the immature immune system and underdeveloped skin barrier function in infants, over 50% of cases experience two or more complications, primarily including peristomal dermatitis, stoma prolapse, stoma necrosis, and mucocutaneous separation. These complications mostly occur between 21 and 40 days postoperatively (9–12). Among them, peristomal dermatitis is the most common complication, with an incidence of 34.04% (13).

Studies have shown that enterostomy-related complications not only significantly increase the risk of infection and prolong hospitalization, but in severe cases, may lead to reoperation or delayed stoma reversal (14–16). More worryingly, increased evidence suggests that enterostomy-related complications are correlated with development retardation in infants (17). A study reported that among infants who underwent enterostomy due to NEC, 89% experienced growth retardation, and 42% met the criteria for severe underweight at the time of stoma closure (1). This might be associated with fluid and electrolyte losses caused by stoma-related complications. Therefore, it is crucial to accurately identify the risk factors for enterostomy-related complications and implement targeted interventions for high-risk infants.

Although some studies have explored the risk factors for enterostomy-related complications in infants, the relevant evidence has significant limitations and controversies. Firstly, the risk factors reported in different studies are inconsistent, with a lack of consensus. Some studies have indicated that younger gestational age, smaller weight at surgery, and shorter stoma duration are potential risk factors for enterostomy-related complications in infants (18, 19). However, other studies have reported that the type of primary disease type (12), emergency surgery (20), and the stoma site (21) are significantly correlated with enterostomy-related complications in infants. Secondly, the research variables lack comprehensiveness. They are mostly confined to a few baseline characteristics variables (such as gestational age and birth weight), and lack systematic integrated analysis of perioperative indicators (such as stoma site and stoma type) and dynamic biomarkers (such as CRP trends). Finally, the sample sizes of existing studies are generally small (*n* < 100), leading to imprecise estimation of effect sizes. Most of the analytical methods stay at univariate analysis or logistic regression without adjusting for confounding factors, failing to construct comprehensive predictive models with clinical practical value. These deficiencies result in its insufficient clinical applicability. To systematically address these limitations and enhance the predictive ability, this study will use a large-scale, high-quality dataset, adopt a strict variable screening process, widely include potential predictive variables indicated by literature reports and clinical experience, and construct and validate a prediction model for enterostomy-related complications in infants. To identify high-risk infants early and implement targeted interventions, such as dynamically monitoring CRP levels to guide anti-inflammatory management and regulate excessive inflammatory responses, initiating enhanced nursing protocols in advance (e.g., hydrocolloid dressings for protective measures) to reduce the risk of skin damage, and formulating individualized nutritional support strategies to optimize nutrient and electrolyte supplementation, thereby enhancing the infants’ resistance. Thereby realizing the transformation from “empirical care” to “data-driven precision intervention,” and ultimately reducing the incidence of complications, minimizing reoperation, shortening the ostomy reversal time, and improving the growth and development outcomes.

## 2 Methods

### 2.1 Study population

Infants who underwent enterostomy for a variety of gastrointestinal and other abdominal conditions from November 2021 to June 2024 were retrospectively included as study subjects. The inclusion criteria were as follows: (1) infants who underwent enterostomy; (2) age <1 year. The exclusion criteria were: (1) complicated with other serious diseases such as congenital heart disease and persistent pulmonary hypertension of the newborn; (2) the discharge outcome is unknown; (3) automatic discharge without completing the treatment process; (4) incomplete medical records. This study was performed in line with the principles of the Declaration of Helsinki. Approval was granted by the Ethics Committee of the Sichuan University West China Second Hospital [YXKY(2024332)]. All data were encoded and used anonymously in this study.

### 2.2 Sample size calculation

The sample size was estimated using the events per variable (EPV) method (22), a widely accepted method in statistical analyses. In our study, the incidence of enterostomy-related complications was 39.35%, and we intended to include 3 predictor variables for model development. The EPV was set at 20 to ensure model stability and statistical power. The required sample size was calculated via the following formula:


$$\text{Sample Size}=\frac{\text{Number of Variables}\times \mathrm{EPV}}{\text{Incidence Rate}}=(3\times 20)/(39.35\%)=153$$

### 2.3 Data collection

The following data were collected via the electronic medical record system, (1) Baseline characteristics including gender, gestational age, birth weight, and delivery mode. (2) Preoperative data including age and weight at surgery, primary disease type, complicated disease, emergency surgery or not, preoperative antibiotics, preoperative total protein value, preoperative prealbumin value, preoperative CRP value and preoperative platelets count. (3) Surgery data including stoma site, stoma type, surgical modality, surgical duration, and intestinal tube resection or not. (4) Postoperative data including feeding initiation time, time to achieve full enteral nutrition, postoperative total protein value, postoperative prealbumin value, postoperative CRP value, postoperative platelet count, postoperative peak CRP value, duration of postoperative high-level CRP, blood transfusion or not, and length of stay in neonatal intensive care unit (NICU). The dataset of our study is complete, with no missing values requiring imputation or exclusion.

### 2.4 Variable definitions

Surgeons and enterostomal therapists collaboratively conduct standardized assessment of enterostomy-related complications using validated evaluation tools, such as the DET scale, Criteria of enterostomy complications: classification and grading (2023 edition). These complications include peristomal dermatitis, mucocutaneous separation, stoma prolapse, stoma retraction, stoma depression, stoma stenosis, parastomal hernia, stoma edema, stoma bleeding, parastomal fistula, stoma necrosis, and high-output stoma. In this study, stoma-related complications were defined as the independent occurrence or combined presence of these complications (23). Primary disease refer to conditions characterized by intestinal structural abnormalities or severe functional disorders that necessitate enterostomy to establish an abdominal excretory pathway. Based on the presence or absence of concurrent infection, they are categorized into Infectious diseases (e.g., NEC) and non-infectious diseases (e.g., Hirschsprung’s disease, anal atresia). Complicated disease refer to pre-existing other diseases in neonates prior to enterostomy, including neonatal pneumonia, neonatal respiratory distress syndrome, neonatal sepsis, neonatal anemia, neonatal hyperbilirubinemia, patent ductus arteriosus, etc. Surgical modality were classified as laparotomy and laparoscope surgery based on the extent of surgical trauma and access technique. Stoma type were classified as single-lumen stomas and double-lumen stomas based on whether the bowel is divided and the number of openings. CRP is the most sensitive indicator for the ancillary diagnosis of inflammatory diseases and is typically below 10 mg/L, therefore CRP > 10 mg/L was considered to be abnormal in this study (24).

### 2.5 Statistical analysis

The statistical analyses were performed using SPSS software (version 26.0; IBM SPSS, Inc., Armonk, NY) and R software (version 4.2.1; R Foundation for Statistical Computing, Vienna, Austria). The normal distribution variables were expressed as the mean ± SD, and tested by student’s *t*-test. The skewed distribution variables were expressed as the median and Inter-Quartile Range, and tested by the Mann–Whitney U test. Categorical variables were presented as percentages, and tested by the chi-squared test or Fisher’s exact test. The variance inflation factor (VIF) was used to diagnose the multicollinearity of independent variables, and the collinearity indicator (VIF > 10) was excluded. Then, the variables with statistical significance (*p* < 0.05) in the univariate analysis were included in the logistic regression to analyze the risk factors of postoperative complications related to enterostomy in infants and construct a nomogram prediction model. The cor() function from the “stats” package in R software (version 4.4.3; R Foundation for Statistical Computing, Vienna, Austria) was used to perform correlation analysis on the independent variables, which allows for the selection of either Pearson or Spearman methods depending on the data characteristics and research question. The resulting coefficients were then visualized using heatmaps, enabling customizable and intuitive graphical representations of the relationships. The Bootstrap repeated sampling method (*B* = 2000) was adopted to conduct internal validation of the prediction model, so as to evaluate the robustness of the model parameters and the generalizability of the prediction performance. The predictive performance was evaluated by the C index, calibration curve, area under the ROC curve, and Hosmer-Lemeshow goodness of fit test. The clinical application value was evaluated by DCA, and *p* < 0.05 was considered statistically significant.

## 3 Results

### 3.1 General characteristics

**Figure 1:**
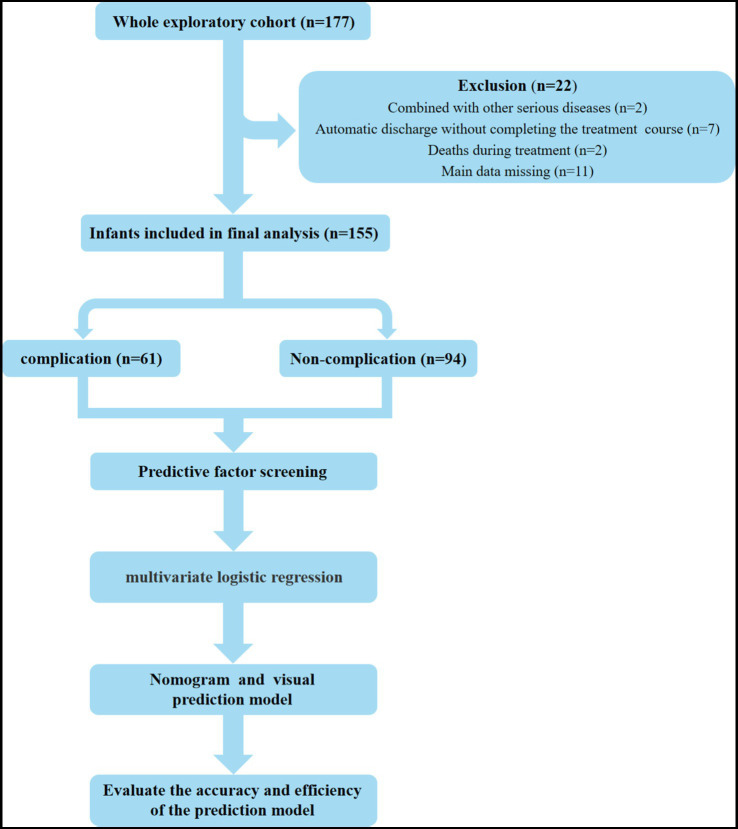
Study flow chart.

**Figure 2:**
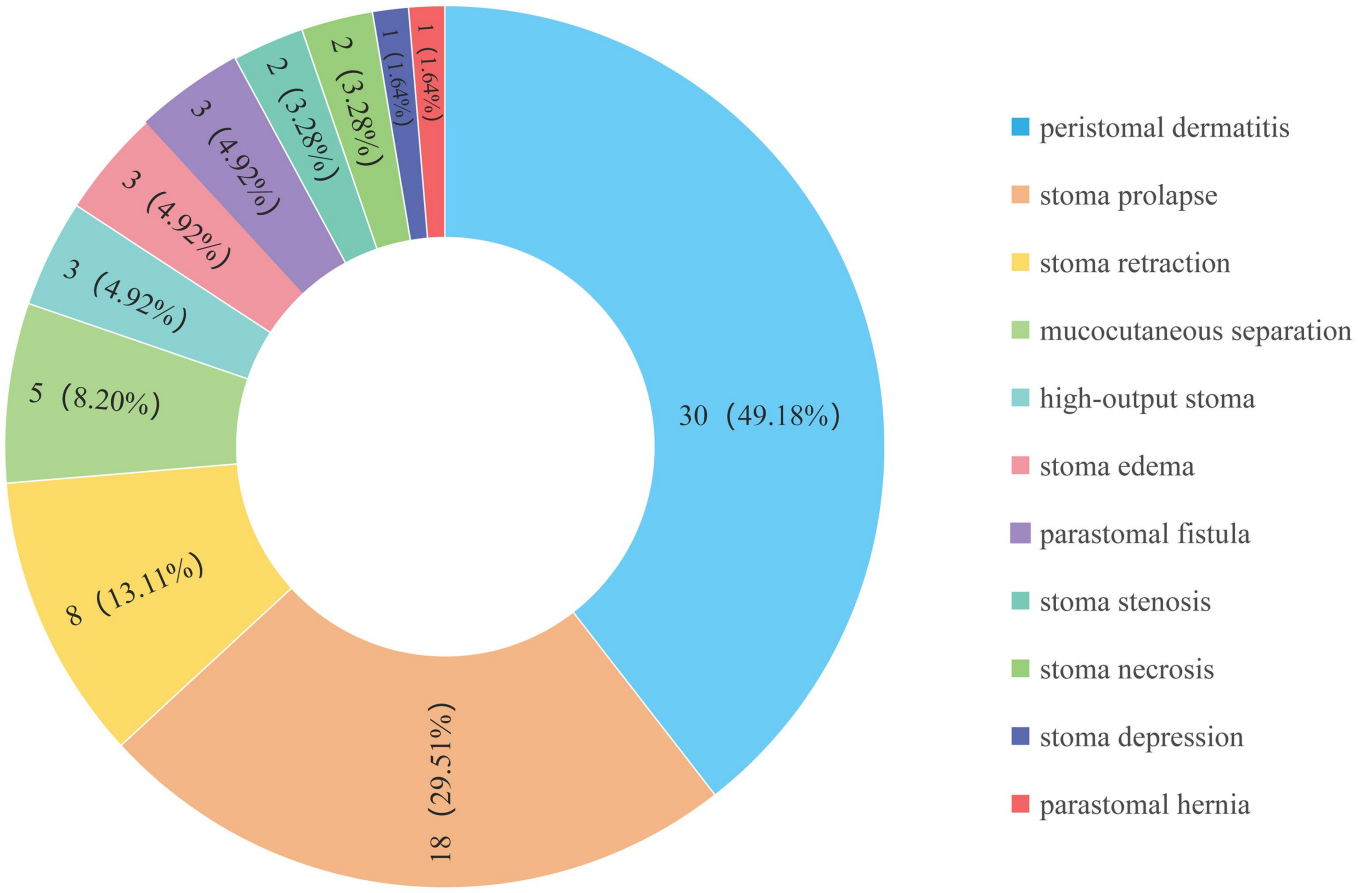
Pie chart of enterostomy-related complications in infants.

This study included a total of 155 infants who underwent enterostomy (Figure 1), consisting of 90 males (58.06%) and 65 females (41.94%). The mean gestational age at birth was 36.33 ± 0.29 weeks, and the mean birth weight was 2582.76 ± 69.36 g. The infants were divided into the complication group (*n* = 61) and the non-complication group (*n* = 94), the incidence of enterostomy-related complications was 39.35% (Figure 2). The descriptive analysis of the study population is presented in Table 1.

**Table 1:** Descriptive analysis of the study populations and univariate analysis.

Variable	Complication group (*n* = 61)	Non-complication group (*n* = 94)	Statistical value	*p* value
Primary disease type [*n* (%)]			***χ***^**2**^ **= 7.260**	**0.007**
Infectious diseases	16 (26.23%)	45(47.87%)		
Non-infectious diseases	45 (73.77%)	49(52.13%)		
Complicated diseases [*n* (%)]			*χ*^2^ = 3.524	0.06
Yes	9 (14.75%)	26(27.66%)		
No	52 (85.25%)	68(72.34%)		
Gender [*n* (%)]			*χ*^2^ = 3.260	0.071
Male	30 (49.18%)	60(63.83%)		
Female	31 (50.82%)	34(36.17%)		
Gestational age [*n* (%)]			***χ***^**2**^ **= 5.438**	**0.02**
Preterm infant	37 (60.66%)	39(41.49%)		
Term infant	24 (39.34%)	55(58.51%)		
Birth weight (g)	2300.00 (1530.00, 2950.00)	3017.50 (2152.50, 3355.00)	***Z* = −3.672**	**<0.001**
Delivery mode [*n* (%)]			*Z* = 0.106	0.745
Vaginal delivery	21 (34.43%)	30 (31.91%)		
Cesarean delivery	40 (65.57%)	64 (68.09%)		
Weight at surgery (g)	2512.62 ± 94.90	2964.36 ± 83.99	***T* = 3.494**	**0.001**
Age at surgery (d)	16.00 (4.00, 31.50)	6.00 (2.20, 25.00)	***Z* = −2.015**	**0.044**
Feeding initiation time (d)	5.00 (3.50, 7.00)	4.00 (2.75, 6.00)	***Z* = −1.971**	**0.049**
Time to achieve full enteral nutrition (d)	24.00 (15.00, 40.00)	17.00 (13.00, 26.00)	***Z* = −2.880**	**0.004**
Preoperative antibiotics [*n* (%)]			Fisher’s exact test	**0.024**
Unrestricted-use grade	2 (3.28%)	3 (3.19%)		
Restricted-use grade	34 (55.74%)	71 (75.53%)		
Special-use grade	25 (40.98%)	20 (21.28%)		
Emergency surgery [*n* (%)]			*χ*^2^ = 0.058	0.810
Yes	28 (45.90%)	45 (47.87%)		
No	33 (54.10%)	49 (52.13%)		
Stoma site [*n* (%)]			***χ***^**2**^ **= 17.028**	**<0.001**
Colonic stoma	6 (9.84%)	38 (40.43%)		
Small intestine stoma	55 (90.16%)	56 (59.57%)		
Stoma type [*n* (%)]			***χ***^**2**^ **= 9.017**	**0.003**
Single-lumen	26 (42.62%)	19 (20.21%)		
Double-lumen	35 (57.38%)	75 (79.79%)		
Intestinal tube resection [*n* (%)]			***χ***^**2**^ **= 3.875**	**0.049**
Yes	24 (39.34%)	23 (24.47%)		
No	37 (60.66%)	71 (75.53%)		
Surgical modality [*n* (%)]			Fisher’s exact test	0.765
Laparotomy	57 (93.44%)	86 (91.49%)		
Laparoscope	4 (6.56%)	8 (8.51%)		
Surgical duration (min)	55.00 (47.00, 80.00)	59.00 (45.00, 90.00)	*Z* = −0.548	0.584
Preoperative total protein value (g/L)	46.20 (39.95, 52.60)	50.20 (45.45, 54.53)	***Z* =** −**2.723**	**0.006**
Preoperative prealbumin value (g/L)	53.00 (32.00, 82.50)	59.00 (40.75, 80.00)	*Z* = −1.352	0.176
Preoperative CRP value (mg/L)	14.20 (1.70, 49.30)	2.90 (0.50, 38.93)	***Z* =** −**2.283**	**0.022**
Preoperative platelets count (10^9^/L)	231.00 (133.00, 359.50)	258.00 (197.00, 354.00)	*Z* = −1.537	0.124
Postoperative total protein value (g/L)	42.90 (39.10, 48.50)	45.30 (39.90, 49.73)	*Z* = −1.522	0.128
Postoperative prealbumin value (g/L)	65.00 (34.00, 89.00)	69.00 (39.50, 112.00)	*Z* = −1.058	0.29
Postoperative CRP value (mg/L)	73.50 (31.20, 98.65)	44.40 (14.78, 93.15)	*Z* = −1.883	0.06
Postoperative platelet count (10^9^/L)	180.00 (84.50, 262.50)	197.00 (142.25, 303.75)	***Z* =** −**1.987**	**0.047**
Postoperative peak CRP value (mg/L)	93.30 (50.20, 129.65)	71.10 (27.85, 126.23)	*Z* = −1.601	0.109
Duration of postoperative high-level CRP (d)	7.00 (4.00, 11.50)	5.00 (3.00, 7.00)	***Z* =** −**3.105**	**0.002**
Blood transfusion [*n* (%)]			***χ***^**2**^ **= 6.603**	**0.01**
Yes	16 (26.23%)	44 (46.81%)		
No	45 (73.77%)	50 (53.19%)		
Length of stay in NICU (d)	37.00 (21.50, 68.50)	25.00 (19.00, 40.25)	***Z* =** −**3.191**	**0.001**

The bold values indicates that the results are statistically significant.

### 3.2 Correlation heatmap of the predictor variables

**Figure 3:**
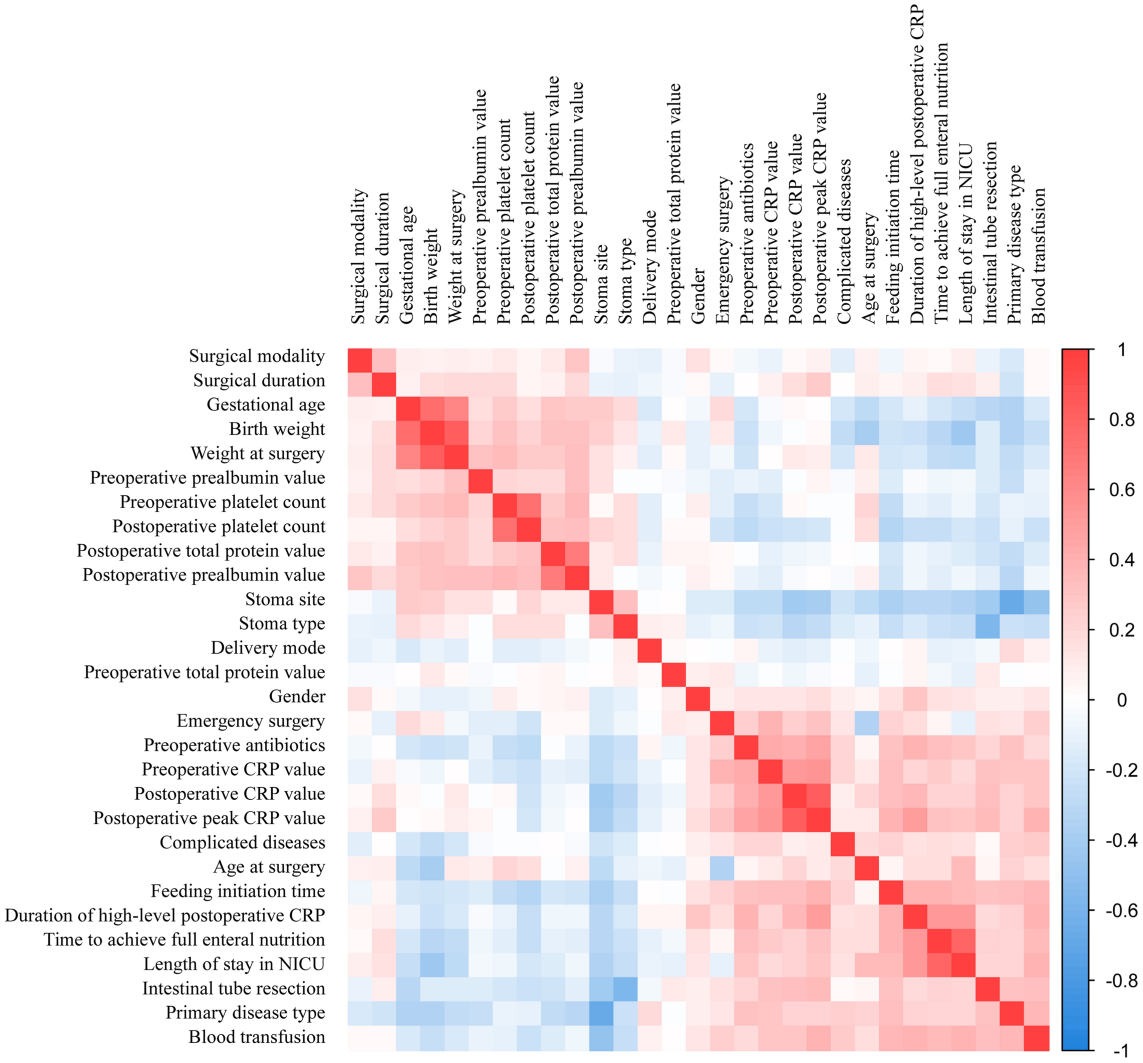
Correlation matrix of variables.

The correlation matrix of variables can be used to illustrate the relationships between various predictor variables. The darker the color of the matrix, the stronger the correlation between variables, with the deep blue and red areas representing strong negative and positive correlations. The correlation matrix in this study predominantly consists of light-colored cells, indicating that the interactions between variables are relatively weak (as shown in Figure 3). For example, there was a strong positive correlation between time to achieve full enteral nutrition and length of stay in NICU, indicating that prolonged NICU stays were associated with slower establishment of enteral nutrition. Similarly, birth weight showed a strong positive correlation with weight at surgery, indicating that infants with higher birth weights tended to maintain greater body mass during surgical interventions. These patterns highlight how the study variables are interconnected and influence each other, reinforcing the need to factor in these relationships during our analysis.

### 3.3 Univariate analysis

In the univariate analysis, the primary disease type, gestational age, birth weight, weight at surgery, age at surgery, feeding initiation time, time to achieve full enteral nutrition, preoperative antibiotics, stoma site, stoma type, intestinal tube resection, preoperative total protein value, preoperative CRP value, postoperative platelet count, duration of postoperative high-level CRP, blood transfusion, and length of stay in NICU were all significantly correlated with a higher incidence of enterostomy-related complications (*p* < 0.05) (Table 1).

Collinearity assessment was performed on variables with statistical significance in univariate analysis. Results showed high multicollinearity between birth weight and weight at surgery (VIF > 10), while other variables demonstrated no significant multicollinearity (1.055 ≤ VIF ≤ 5.466). Therefore, birth weight (larger VIF value) was excluded in the Logistic regression analysis to enhance the accuracy of the model.

### 3.4 Multivariate analysis

The fifteen variables with *p* < 0.05 and VIF < 10 in the univariate analysis as well as the weight at surgery were taken as independent variables, and whether complications occurred after surgery was taken as the dependent variable for binary logistic regression analysis. In the multivariate analysis, with results reported as odds ratio (95% CI), weight at surgery (OR = 0.999, 95%CI: 0.999–1.000; *p* = 0.044), duration of postoperative high-level CRP (OR = 1.081, 95%CI: 1.001–1.167; *p* = 0.048), and the stoma site (OR = 6.405, 95%CI: 1.647–24.916; *p* = 0.007) were independently associated with the risk of complications after enterostomy in infants (Table 2).

**Table 2:** Multivariate logistic regression analysis of factors associated with complications after enterostomy in infants.

Variable		*β*	SE	Wald*χ*^2^	*p*	OR	95%CI
Primary disease type		0.509	0.563	0.816	0.366	1.663	0.551 ~ 5.019
Gestational age		−0.341	0.58	0.346	0.556	0.711	0.228 ~ 2.216
Weight at surgery		**−0.001**	**0.0004**	**4.039**	**0.044**	**0.999**	**0.999 ~ 1.000**
Age at surgery		−0.003	0.012	0.084	0.772	0.997	0.974 ~ 1.020
Feeding initiation time		−0.072	0.089	0.654	0.419	0.931	0.782 ~ 1.108
Time to achieve full enteral nutrition		0.001	0.015	0.003	0.956	1.001	0.971 ~ 1.031
Preoperative antibiotics	Restricted-use grade	0.63	1.274	0.244	0.621	1.877	0.155 ~ 22.801
	Special-use grade	−0.24	0.509	0.222	0.638	0.787	0.290 ~ 2.136
Stoma site		**1.857**	**0.693**	**7.18**	**0.007**	**6.405**	**1.647 ~ 24.916**
Stoma type		0.83	0.503	2.721	0.099	2.294	0.855 ~ 6.151
Intestinal tube resection		0.117	0.553	0.045	0.832	1.124	0.381 ~ 3.322
Preoperative total protein value		−0.039	0.033	1.379	0.24	0.962	0.902 ~ 1.026
Preoperative CRP value		−0.004	0.005	0.528	0.468	0.996	0.987 ~ 1.006
Postoperative platelet count		−0.0001	0.002	0.003	0.953	1.000	0.996 ~ 1.003
Duration of postoperative high-level CRP		**0.077**	**0.039**	**3.922**	**0.048**	**1.081**	**1.001 ~ 1.167**
Blood transfusion		0.273	0.492	0.308	0.579	1.314	0.501 ~ 3.446
Length of stay in NICU		0.0003	0.013	0.0004	0.984	1	0.974 ~ 1.027
Constant		1.695	2.219	0.583	0.445	5.447	—

Variable assignment (1) primary disease type: Non-infectious diseases = 0, Infectious diseases = 1; (2) gestational age: preterm infant = 0, term infant = 1; (3) preoperative antibiotics: unrestricted-use grade = 0, restricted-use grade = 1, special-use grade = 2; (4) stoma site: colonic stoma = 0, small intestine stoma = 1; (5) stoma type: single-lumen = 0, double-lumen = 1; (6) intestinal tube resection: No = 0, Yes = 1; (7) blood transfusion: No = 0, Yes = 1. The bold values indicates that the results are statistically significant.

### 3.5 Development of risk prediction model

**Figure 4:**
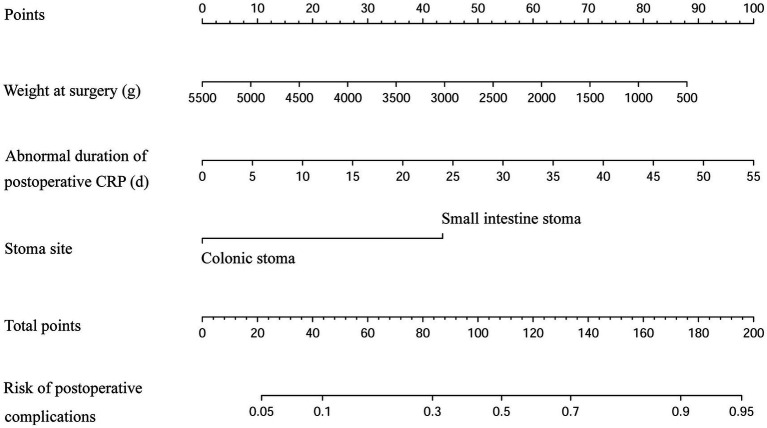
Nomogram of the postoperative enterostomy-related complication risks. (The scores of each variable in the nomogram are summed, and the projection of the total score onto the risk axis signifies the predicted probability. The greater the total score, the higher the risk of postoperative complications for the infant).

Three statistically significant variables in logistic regression analysis were used to construct regression equations. Weight at surgery, duration of postoperative high-level CRP, and small intestinal stoma were represented by *X*_1_, *X*_2_, and *X*_3_, respectively. The regression equation was developed as follows: Logit (*p*) = 1.695–0.001*X_1_ + 0.077*X_2_ + 1.857*X_3_. The probability of complications decreased by 0.074% when the weight at surgery increased by 1 g, the probability of complications increased by 8.10% when the duration of postoperative high-level CRP increased by 1 day, and the probability of complications in infants with a small intestinal stoma was 6.405 times higher than that in infants with a colonic stoma. The nomogram model was constructed using the above significant predictors, as shown in Figure 4. The C-index of this nomogram was 0.761 (95%CI: 0.686–0.836). When the weight at surgery decreased, the duration of postoperative high-level CRP increased, and the stoma site was the small intestine stoma, the risk score of the nomogram increased accordingly, indicating that the nomogram can accurately predict the risk of enterostomy complications.

### 3.6 Evaluation of the prediction model

**Figure 5:**
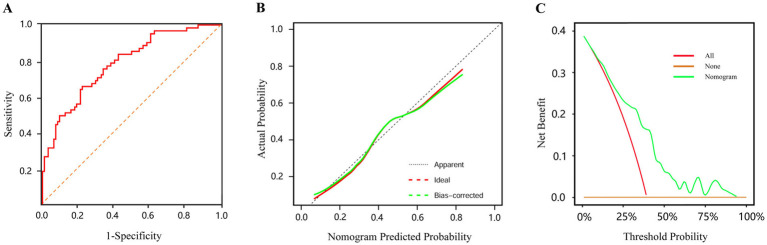
The evaluation of prediction model **(A)** ROC curve for predicting postoperative complications after enterostomy in infants. **(B)** Calibration curve of the nomogram prediction model (the *x*-axis shows the predicted probability of the nomogram, and the *y*-axis represents the observed probability. The red line shows the ideal curve, and the green line shows the bias-corrected curve). **(C)** DCA of the nomogram prediction model (the none curve signifies that all infants do not receive any intervention and the net benefit is 0. The all curve signifies that all infants receive the intervention and the slope of the curve is the net benefit. The nomogram curve signifies the net benefit of the nomogram model in predicting the risk of postoperative complications in infants).

This study retrospectively analyzed the clinical data of infants with enterostomy, established a nomogram model for predicting the risk of complications, and verified the accuracy of the prediction model through various methods. The Bootstrap method with 2000 resampling iterations was used to evaluate the model’s discriminative ability and calibration. The results showed that the AUC was 0.784 (95%CI: 0.712–0.857), the Youden index was 0.433, the sensitivity was 65.60%, and the specificity was 77.70%, indicating that the model had good predictive performance (Figure 5A). The bias-corrected curve approached the ideal curve, and the Hosmer-Lemeshow test showed no statistically significant difference between the actual risk and the predicted risk of the model (*χ*^2^ = 6.389, *p* = 0.604), suggesting that the model had a good fitting effect (Figure 5B). Furthermore, The DCA showed that the nomogram had better potential for clinical application when the threshold probability was 0.05–0.93 (Figure 5C).

## 4 Discussion

Temporary stoma formation is a crucial means of surgical treatment for a variety of intestinal diseases in infants, playing a crucial role in saving lives and contributing to the cure of the disease (25). Despite advancements in surgical techniques and perioperative management, with the incidence of complications decreasing from 42.47% ~ 68.00% and then to 17.28% ~ 59.49%, complication incidence still remains at a relatively high level (7, 8, 13, 18). The overall incidence of postoperative complications in this study was 39.35% and peristomal dermatitis was found to be the most common complication, with an incidence of 19.35%. The results were consistent with other reported data worldwide (26). Through multivariate logistic regression analysis, we finally identified that the weight at surgery, stoma site, and the duration of postoperative high-level CRP were the most key predictors. Based on these factors, we developed a nomogram model to predict enterostomy-related complications in infants at an early stage. To our knowledge, this is the first prediction model established for enterostomy-related complications in infants.

Regarding the weight at surgery, this study found that the greater the weight at surgery, the lower the risk of complications in infants (OR = 0.999, 95%CI: 0.999 ~ 1.000, *p* = 0.044), which is consistent with the research results of Aguayo et al. (18) (*p* = 0.024) and Lee et al. (19) (*p* = 0.014). Low-weight infants are underdeveloped, with relatively weak skin barrier function. They are more vulnerable to the invasion of various pathogenic microorganisms, which significantly increases the risks of postoperative complications (27). Notably, although some studies have shown that low birth weight is also a risk factor for enterostomy-related complications in infants (28, 29), our study did not find a significant association between them (*p* > 0.05). Further analysis revealed that although weight is an important indicator reflecting the growth and immune system maturation of infants, due to disease-specific characteristics and clinical constraints, not all diseases are suitable for immediate surgical treatment during initial stages. This temporal discrepancy leads to significant discrepancy between the weight at surgery and the birth weight. Therefore, compared with the birth weight, which is a static indicator, the weight at surgery can more dynamically reflect the physiological status of infants during the perioperative period.

The stoma at the small bowel was identified as the most significant risk factor for enterostomy-related complications in infants (OR = 6.405, 95%CI: 1.647–24.916, *p* = 0.007), and the complication incidence (49.55%) was significantly higher than that of colostomy (13.64%). Tan and Deogracias (3) also supported this result. Peristomal dermatitis was the most common complication after enterostomy in infants, and 90% of peristomal dermatitis was located in small bowel stoma cases, while colostomies cases were rare. This may be related to the fact that the small intestinal juice is rich in various digestive enzymes and has weakly alkaline properties. It can easily erode the peristomal skin, leading to skin redness, swelling, ulceration, erosion and other symptoms. In severe cases, the entire skin layer may even peel off (30). Steinhagen et al. (31) also pointed out that the excreta from small intestine stomas were highly corrosive, and prolonged maceration will damage the skin integrity around the stoma, which was more likely to induce infection.

The results also revealed that the incidence of stomas prolapse was second only to peristomal dermatitis (accounting for 29.51% of all cases with complications), and all cases of stoma prolapse occurred in infants with small intestinal stomas, which is comparable with the previously reported proportion of 19–42% in the literature (18, 25). Related studies have shown that both the small intestinal wall and the colonic wall are composed of the mucosa, submucosa, muscular layer, and serosa. However, the normally distended small intestinal wall (0.3 cm–0.5 cm) is thinner than the colonic wall (0.5 cm–0.8 cm), which may predispose to higher stoma prolapse risk (32). In addition, the small intestine is an intraperitoneal organ with greater mobility. When the intra-abdominal pressure increases due to an infant’s crying, the axial movement of the small intestine may also be a contributing factor to stoma prolapse (33). However, not all studies supported this conclusion. The colonic contents are a semi-solid material, and their movement speed is faster than the peristaltic waves of the ileum. Geng et al. (34) suggested that this phenomenon makes poststomal colonic prolapse more likely to occur than ileal prolapse. Therefore, regarding the result of the higher incidence of small intestinal stoma prolapse, it still requires the support of high-quality literature evidence.

Furthermore, all cases of HOS also occurred in infants with small intestinal stomas, accounting for 4.92% of all cases with complications. Stoop et al. (35) hold that the small intestine is relatively rich in water content, when intestinal continuity is disrupted, a large amount of intestinal juice will be discharged through the stoma, thereby inducing high-output diarrhea. Another possible explanation is that the contents of the ileum are more concentrated and more easily diluted than those of the colon, which results in excessive output (36). This study synthesized existing evidence and further confirmed that infants with small intestine stomas face significantly higher risks of complications. These findings establish an essential evidence base for the stratified management based on the stoma site in clinical practice.

The longer duration of postoperative high-level CRP was also found to be a predictive factor in this study. (OR = 1.081, 95%CI: 1.001 ~ 1.167, *p* = 0.048). Although some scholars have proposed that the preoperative or postoperative CRP levels can be used as predictive indicators for infectious complications after enterostomy, few studies reported the relation between the duration of abnormal CRP levels and enterostomy-related complications in infants (37–39). This is a newly discovered risk factor in our study. CRP is an infection-related biomarker and rises sharply when the body is infected by inflammation or suffers from tissue damage (37). It is a key indicator for the diagnosis of infectious or inflammatory diseases. However, the exact mechanism by which it predicts postoperative infectious complications remains incompletely understood (40, 41). Niu et al. (42) hold that continuously elevated inflammatory factors can induce wound healing disorders characterized by pathological fibrous hyperplasia, complicate wound healing, and thus significantly increase the risk of complications. Another study (43) showed that high levels of inflammatory factors can suppress the synthesis of collagen, decrease both the concentration and activity of fibroblasts, cause tissue degeneration, and reduce peristomal skin support for the intestine, thereby leading to complications such as parastomal hernia. However, Zhu et al. (41) believed that the mechanism by which high-level CRP increases the risk of postoperative complications involves the synergistic effects of impaired healing and impaired immune response. It is worth noting that although we found a significant correlation between the duration of postoperative high-level CRP and complication risks, the critical value remains uncertain. Moreover, previous studies explored only the peak CRP before or after surgery, which is flawed because it cannot determine whether the abnormal CRP is due to postoperative stress, inflammation, or other reactions. Future studies should further explore and validate the application value of the duration of abnormal postoperative CRP in predicting enterostomy complications.

## 5 Study limitations

We identified risk factors of enterostomy-related complications and constructed an effective risk prediction model in infants with stomas. There are still several limitations to our study that must be acknowledged. Firstly, this study was a single-center retrospective investigation with a relatively small sample size, which may result in selection bias. Secondly, although this study included the most valuable predictors for analysis, other unmeasured or unknown confounding factors such as stoma height and incision diameter may have a potential impact on this prediction model. Thirdly, there are inherent differences in surgical practices, patient populations, and postoperative care protocols across institutions. Fourthly, some predictor variables are postoperative indicators, which are subject to time lag effects, thus limiting their early predictive utility. Finally, the predictive model was only internally validated, and its performance still requires external validation with datasets from other research centers.

## 6 Conclusion

In this study, we found that smaller weight at surgery, small intestinal stoma, and longer duration of postoperative high-level CRP were independent risk factors for postoperative complications related to enterostomy in infants. The nomogram model constructed based on the above factors exhibited good discrimination, calibration, and clinical validity. At the same time, the nomogram model was capable of visualizing clinical indicators to assess the occurrence probability of complications, thereby providing a basis for medical staff to identify infants with a relatively high risk of postoperative complications early and adopt targeted preventive measures.

## Data Availability

The raw data supporting the conclusions of this article will be made available by the authors, without undue reservation.

## References

[1] Chong C van Druten J Briars G Eaton S Clarke P Tsang T. Neonates living with enterostomy following necrotising enterocolitis are at high risk of becoming severely underweight. Eur J Pediatr. (2019) 178:1875–81. doi: 10.1007/s00431-019-03440-6, PMID: 31522315 PMC6892362

[2] Liu Z Zhang Y Li S. Risk factors of enterostomy in neonates with Hirschsprung disease. Int J Colorectal Dis. (2022) 37:1127–1132. doi: 10.1007/s00384-022-04151-5 PMID: 35449241

[3] Tan AYE Deogracias JMC. Major stoma complications in pediatric patients in a tertiary hospital in a low-middle-income country: a retrospective cohort study. Pediatr Surg Int. (2024) 40:208. doi: 10.1007/s00383-024-05791-0, PMID: 39044020

[4] Emil S Nguyen T Sills J Padilla G. Meconium obstruction in extremely low-birth-weight neonates: guidelines for diagnosis and management. J Pediatr Surg. (2004) 39:731–7. doi: 10.1016/j.jpedsurg.2004.01.027, PMID: 15137008

[5] Struijs MC Sloots CE Hop WC Tibboel D Wijnen RM. The timing of ostomy closure in infants with necrotizing enterocolitis: a systematic review. Pediatr Surg Int. (2012) 28:667–72. doi: 10.1007/s00383-012-3091-9, PMID: 22526553 PMC3376257

[6] Zani A Lauriti G Li Q Pierro A. The timing of stoma closure in infants with necrotizing enterocolitis: a systematic review and meta-analysis. Eur J Pediatr Surg. (2017) 27:007–11. doi: 10.1055/s-0036-1587333 27522125

[7] Singhal G Ramakrishnan R Goldacre R Battersby C Hall NJ Gale C. UK neonatal stoma practice: a population study. Arch Dis Child Fetal Neonatal Ed. (2024) 110:79–84. doi: 10.1136/archdischild-2024-327020 38897635 PMC11671890

[8] O'Connor A Sawin RS. High morbidity of enterostomy and its closure in premature infants with necrotizing enterocolitis. Arch Surg. (1998) 133:875–80. doi: 10.1001/archsurg.133.8.875, PMID: 9711962

[9] Talbot LJ Sinyard RD Rialon KL Englum BR Tracy ET Rice HE. Influence of weight at enterostomy reversal on surgical outcomes in infants after emergent neonatal stoma creation. J Pediatr Surg. (2017) 52:35–9. doi: 10.1016/j.jpedsurg.2016.10.015, PMID: 27916444

[10] Vogel I Eeftinck Schattenkerk LD Venema E Pandey K de Jong JR Tanis PJ. Major stoma related morbidity in young children following stoma formation and closure: a retrospective cohort study. J Pediatr Surg. (2022) 57:402–6. doi: 10.1016/j.jpedsurg.2021.11.021, PMID: 34949444

[11] Salvadalena GD. The incidence of stoma and peristomal complications during the first 3 months after ostomy creation. J Wound Ostomy Continence Nurs. (2013) 40:400–6. doi: 10.1097/WON.0b013e318295a12b, PMID: 23820472

[12] Wolf L Gfroerer S Fiegel H Rolle U. Complications of newborn enterostomies. World J Clin Cases. (2018) 6:1101–10. doi: 10.12998/wjcc.v6.i16.1101, PMID: 30613668 PMC6306644

[13] Chen SY Grisotti G Mack SJ Walther AE Chapman RL Falcone RA Jr. A multi-institutional study comparing stoma location in neonates with intestinal perforation. J Surg Res. (2024) 297:56–62. doi: 10.1016/j.jss.2024.01.031, PMID: 38432084

[14] Segal I Kang C Albersheim SG Skarsgard ED Lavoie PM. Surgical site infections in infants admitted to the neonatal intensive care unit. J Pediatr Surg. (2014) 49:381–4. doi: 10.1016/j.jpedsurg.2013.08.001, PMID: 24650461 PMC5756080

[15] Hendren S Hammond K Glasgow SC Perry WB Buie WD Steele SR. Clinical practice guidelines for ostomy surgery. Dis Colon Rectum. (2015) 58:375–87. doi: 10.1097/DCR.0000000000000347, PMID: 25751793

[16] Aubert M Buscail E Duchalais E Cazelles A Collard M Charleux-Muller D. Management of adult intestinal stomas: the 2023 French guidelines. J Visc Surg. (2024) 161:106–28. doi: 10.1016/j.jviscsurg.2024.02.002, PMID: 38448363

[17] Rees CM Pierro A Eaton S. Neurodevelopmental outcomes of neonates with medically and surgically treated necrotizing enterocolitis. Arch Dis Child Fetal Neonatal Ed. (2007) 92:F193–8. doi: 10.1136/adc.2006.099929, PMID: 16984980 PMC2675329

[18] Aguayo P Fraser JD Sharp S St Peter SD Ostlie DJ. Stomal complications in the newborn with necrotizing enterocolitis. J Surg Res. (2009) 157:275–8. doi: 10.1016/j.jss.2009.06.005, PMID: 19815238

[19] Lee J Kang MJ Kim HS Shin SH Kim HY Kim EK. Enterostomy closure timing for minimizing postoperative complications in premature infants. Pediatr Neonatol. (2014) 55:363–8. doi: 10.1016/j.pedneo.2014.01.001, PMID: 24582165

[20] Arumugam PJ Bevan L Macdonald L Watkins AJ Morgan AR Beynon J. A prospective audit of stomas-analysis of risk factors and complications and their management. Color Dis. (2003) 5:49–52. doi: 10.1046/j.1463-1318.2003.00403.x, PMID: 12780927

[21] Lockhat A Kernaleguen G Dicken BJ van Manen M. Factors associated with neonatal ostomy complications. J Pediatr Surg. (2016) 51:1135–7. doi: 10.1016/j.jpedsurg.2015.09.026, PMID: 26597393

[22] van Smeden M Moons KG de Groot JA Collins GS Altman DG Eijkemans MJ. Sample size for binary logistic prediction models: beyond events per variable criteria. Stat Methods Med Res. (2019) 28:2455–74. doi: 10.1177/0962280218784726, PMID: 29966490 PMC6710621

[23] The Chinese Ostomy Collaboration Group. Criteria of enterostomy complications: classification and grading (2023 edition). Chin J Gastrointest Surg. (2023) 26:915–21. doi: 10.3760/cma.j.cn441530-20230918-00094, PMID: 37849260

[24] Roy N Ohtani K Matsuda Y Mori K Hwang I Suzuki Y. Collectin CL-P1 utilizes C-reactive protein for complement activation. Biochim Biophys Acta. (2016) 1860:1118–28. doi: 10.1016/j.bbagen.2016.02.012, PMID: 26922829

[25] Massenga A Chibwae A Nuri AA Bugimbi M Munisi YK Mfinanga R. Indications for and complications of intestinal stomas in the children and adults at a tertiary care hospital in a resource-limited setting: a Tanzanian experience. BMC Gastroenterol. (2019) 19:157. doi: 10.1186/s12876-019-1070-5, PMID: 31462228 PMC6714288

[26] Hau EM Meyer SC Berger S Goutaki M Kordasz M Kessler U. Gastrointestinal sequelae after surgery for necrotising enterocolitis: a systematic review and meta-analysis. Arch Dis Child Fetal Neonatal Ed. (2019) 104:F265–73. doi: 10.1136/archdischild-2017-314435, PMID: 29945925

[27] van Zoonen AG Schurink M Bos AF Heineman E Hulscher JB. Ostomy creation in neonates with acute abdominal disease: friend or foe? Eur J Pediatr Surg. (2012) 22:295–9. doi: 10.1055/s-0032-1313346, PMID: 22648187

[28] Bethell G Kenny S Corbett H. Enterostomy-related complications and growth following reversal in infants. Arch Dis Child Fetal Neonatal Ed. (2017) 102:F230–4. doi: 10.1136/archdischild-2016-311126, PMID: 27671835

[29] Chwals WJ Blakely ML Cheng A Neville HL Jaksic T Cox CS Jr. Surgery-associated complications in necrotizing enterocolitis: a multiinstitutional study. J Pediatr Surg. (2001) 36:1722–4. doi: 10.1053/jpsu.2001.27975, PMID: 11685712

[30] Mo J Wendel CS Sloan JA Sun V Hornbrook MC Grant M. Stoma location and ostomy-related quality of life among cancer survivors with ostomies: a pooled analysis. Am J Surg. (2022) 223:963–8. doi: 10.1016/j.amjsurg.2021.09.023, PMID: 34600739 PMC8948094

[31] Steinhagen E Colwell J Cannon LM. Intestinal stomas-postoperative stoma care and peristomal skin complications. Clin Colon Rectal Surg. (2017) 30:184–92. doi: 10.1055/s-0037-1598159, PMID: 28684936 PMC5498169

[32] Ge Z Zhao X Liu Z Yang G Wu Q Wang X. Complications of preventive loop ileostomy versus colostomy: a meta-analysis, trial sequential analysis, and systematic review. BMC Surg. (2023) 23:235. doi: 10.1186/s12893-023-02129-w, PMID: 37568176 PMC10422751

[33] Lei QP. Analysis of risk factors of enterostomy in infants with Hirschsprung's disease. Chongqing Med Univ. (2021). doi: 10.27674/d.cnki.gcyku.2021.000403

[34] Geng HZ Nasier D Liu B Gao H Xu YK. Meta-analysis of elective surgical complications related to defunctioning loop ileostomy compared with loop colostomy after low anterior resection for rectal carcinoma. Ann R Coll Surg Engl. (2015) 97:494–501. doi: 10.1308/003588415X14181254789240, PMID: 26274752 PMC5210131

[35] Stoop TF van Bodegraven EA Ten Haaft BH van Etten‐Jamaludin FS van Zundert SM Lambe C. Systematic review on management of high-output enterostomy in children: an urgent call for evidence. J Pediatr Gastroenterol Nutr. (2024) 78:188–96. doi: 10.1002/jpn3.12043, PMID: 38374570

[36] Yang S Tang G Zhang Y Wei Z du D. Meta-analysis: loop ileostomy versus colostomy to prevent complications of anterior resection for rectal cancer. Int J Color Dis. (2024) 39:68. doi: 10.1007/s00384-024-04639-2, PMID: 38714581 PMC11076370

[37] Kubo T Ono S Ueno H Shinto E Yamamoto J Hase K. Elevated preoperative C-reactive protein levels are a risk factor for the development of postoperative infectious complications following elective colorectal surgery. Langenbeck's Arch Surg. (2013) 398:965–71. doi: 10.1007/s00423-013-1107-0, PMID: 23982867

[38] Xu X Zhou Y Tan Z Huang Y Dong K Gu Y. Risk factors for stoma and incision complications of enterostomy in children with very early-onset inflammatory bowel disease: a prospective cohort study. Eur J Pediatr. (2025) 184:146. doi: 10.1007/s00431-024-05952-2, PMID: 39828783

[39] Abet E Drissi F Couëtte C Jean MH Denimal F Podevin J. Predictive value of inflammatory markers for postoperative recovery following colorectal surgery. Int J Color Dis. (2020) 35:1125–31. doi: 10.1007/s00384-020-03594-y, PMID: 32291509

[40] Bouteloup G Lefevre JH Challine A Voron T O’Connell L Debove C. C-reactive protein values after surgery for inflammatory bowel disease: is it still a good marker for intra-abdominal complication? A retrospective cohort study of 347 procedures. Int J Color Dis. (2022) 37:2347–56. doi: 10.1007/s00384-022-04259-8, PMID: 36243808

[41] Zhu Y Chen J Lin S Xu D. Risk factor for the development of surgical site infection following ileostomy reversal: a single-center report. Updates Surg. (2022) 74:1675–82. doi: 10.1007/s13304-022-01335-0 36002762

[42] Niu N Du S Yang D Zhang L Wu B Zhi X. Risk factors for the development of a parastomal hernia in patients with enterostomy: a systematic review and meta-analysis. Int J Color Dis. (2022) 37:507–19. doi: 10.1007/s00384-021-04068-5, PMID: 35028686

[43] Banerjee DB Vithana H Sharma S Tsang TTM. Outcome of stoma closure in babies with necrotising enterocolitis: early vs late closure. Pediatr Surg Int. (2017) 33:783–6. doi: 10.1007/s00383-017-4084-5, PMID: 28434039

