# Supplementary material for: Risk factors for complications after infantile enterostomy: development of a clinical prediction model
Source: Front Public Health. 2025 Jul 3;13:1566789. doi: 10.3389/fpubh.2025.1566789 (PMC12267257; doi:10.3389/fpubh.2025.1566789)
Supplement: Supplementary file 1 [file Data_Sheet_1.docx]

**Variable selection validation**

**1. Validation method**

Variable selection validation was conducted via the LASSO method. Independent variables with nonzero coefficients in the LASSO regression model were selected and subsequently analyzed via multivariate logistic regression (P < 0.05) to identify potential predictive factors.

**2. Screening for predictive factors**

We employed tenfold cross-validation to determine the optimal tuning parameter λ for the model and detected four variables with nonzero coefficients: weight at surgery, stoma site, stoma type, and duration of postoperative high-level CRP (Supplement Figure S1). These variables exhibited significant predictive power in the model. We assessed potential collinearity issues among these variables, and the VIF of all variables was below 10 (1.039≤VIF≤1.232), indicating no significant collinearity.

**
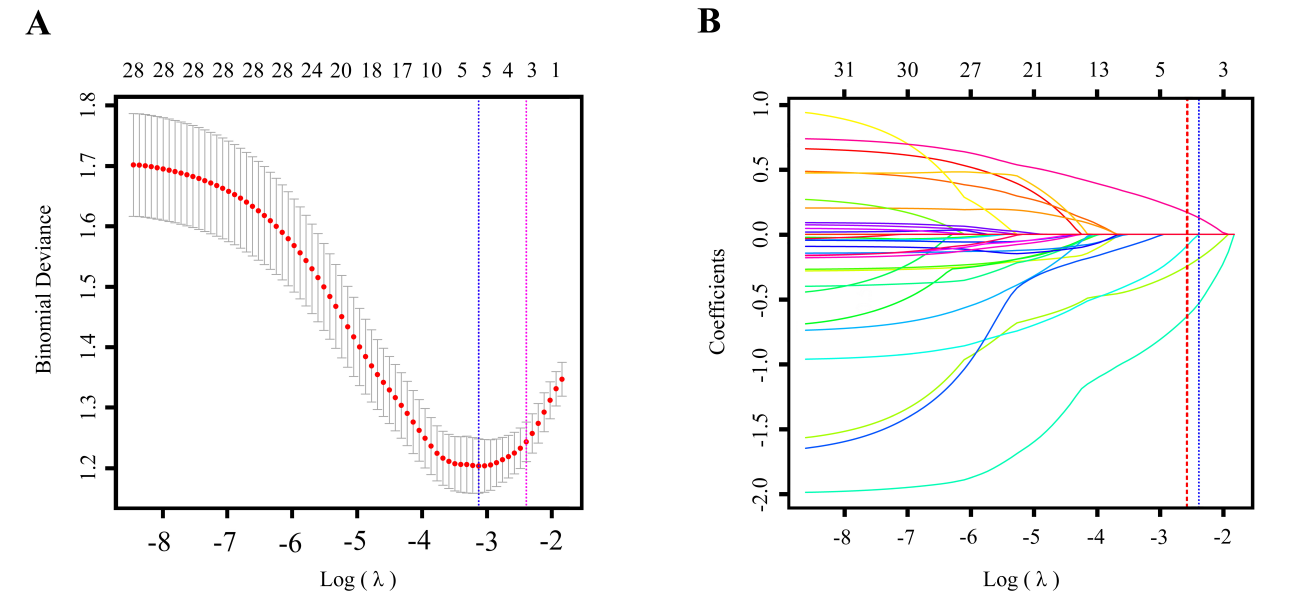
**

**Supplement Figure S1. Variable selection using the LASSO model.** (A) Ten-fold cross-validation for tuning parameter selection in the LASSO model (Each red dot represents the binomial deviance for a specific λ value, with error bars denoting the standard error of the deviance). (B) LASSO coefficient profiles(Different colored lines denote different variables, as Log(λ) changes, the coefficients of most variables approach zero)

**3. Logistic regression analysis**

A multivariate logistic regression model was constructed with these significant indicators and revealed three significant risk factors for complications after enterostomy in infants: weight at surgery (OR=0.999, 95% CI: 0.999-1.000, P=0.003), stoma site (OR=3.589, 95% CI: 1.287-10.008, P=0.015), and duration of postoperative high-level CRP (OR=1.062, 95% CI: 1.001-1.126, P=0.048) (Supplement Table 1). These three factors were statistically significant (*P*<0.05) in predicting complications after enterostomy in infants. The degree of contribution of the three final predictor variables is shown in Supplement Figure S2. The Stoma site had the highest contribution degree (1.278), and the duration of postoperative high-level CRP had the lowest contribution degree (0.461).

**Supplement Table 1 Multivariate Logistic Regression Analysis of risk factors associated with complications after enterostomy in infants**

| **Variable** | **β** | **SE** | **Wald*χ^2^*** | **P** | **OR** | **95%*CI*** |
| --- | --- | --- | --- | --- | --- | --- |
| **Weight at surgery** | -0.001 | 0.00025 | 9.129 | 0.003 | 0.999 | 0.999~1.000 |
| **Stoma site** | 1.278 | 0.523 | 5.966 | 0.015 | 3.589 | 1.287~10.008 |
| **Stoma type** | 0.660 | 0.416 | 2.518 | 0.113 | 1.935 | 0.856~4.371 |
| **Duration of postoperative high-level CRP** | 0.06 | 0.03 | 3.91 | 0.048 | 1.062 | 1.001~1.126 |
| **Constant** | -0.004 | 0.826 | 0.00003 | 0.996 | 0.996 | — |
| **Variable assignment** (1) Stoma site: Colonic stoma=0, Small intestine stoma=1; (2) Stoma type: Single-lumen=0, Double-lumen=1. | | | | | | |


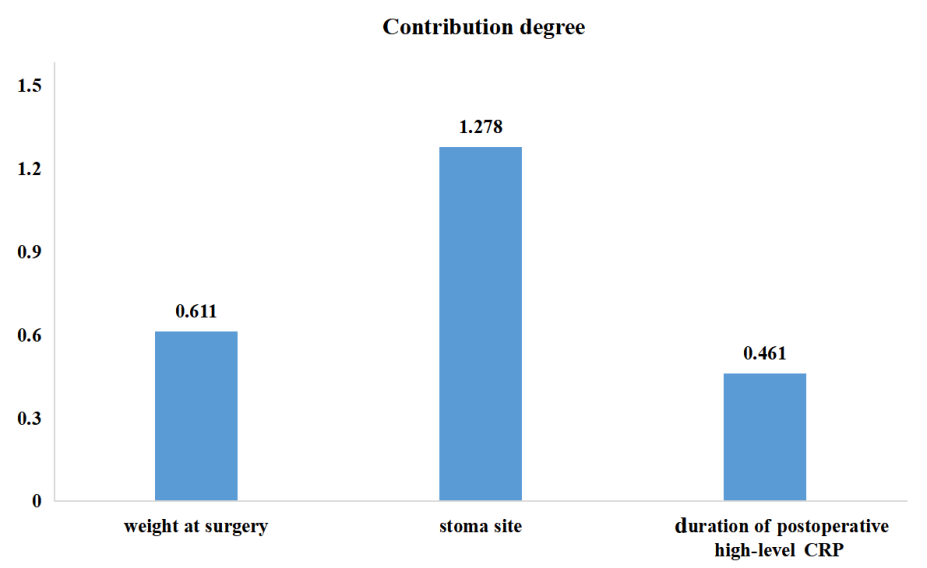


**Supplement Figure S2. The degree of contribution of each factor to the prediction of complications after enterostomy in infants**

**4. Summary**

The reliability of variable selection was validated using LASSO regression. The results showed that the variable selection results of univariate analysis were completely consistent with those of LASSO regression. Both methods identified the same three predictors for model construction: weight at surgery, duration of postoperative high-level CRP, and stoma site. This indicates that the research results have high reliability and research value.
